# Supplementary material for: Interspecific hybrids show a reduced adaptive potential under DNA damaging conditions
Source: Evol Appl. 2020 Dec 15;14(3):758–69. doi: 10.1111/eva.13155 (PMC7980265; doi:10.1111/eva.13155)
Supplement: Supplementary file 1 — Supplementary Material [file EVA-14-758-s001.pdf]

Supplementary Material for:

Interspecific hybrids show a reduced adaptive potential under DNA damaging conditions

Carla Bautista[1,2,3,4], Souhir Marsit[1,2,3,4], Christian R Landry[1,2,3,4,5]

[1] Institut de Biologie Intégrative et des Systèmes (IBIS), Université Laval, Québec, QC, Canada

[2] Département de Biologie|Faculté des Sciences et de Génie, Université Laval, Québec, QC, Canada

[3] Regroupement québécois de recherche sur la fonction, la structure et l'ingénierie des protéines (PROTEO), Université Laval, Québec, QC, Canada

[4] Centre de Recherche en Données Massives (CRDM), Université Laval, Québec, QC, Canada

[5] Département de Biochimie, de Microbiologie et de Bio-informatique|Faculté des Sciences et de Génie, Université Laval, Québec, QC, Canada

Correspondence:

1. Corresponding Author: Carla Bautista (c.bautistarourjc@gmail.com) and Christian R Landry (Christian.landry@bio.ulaval.ca)
2. Other e-mail addresses: Souhir Marsit: (souhir.marsit@gmail.com)

Number of Figures: 8
Number of Tables: 5

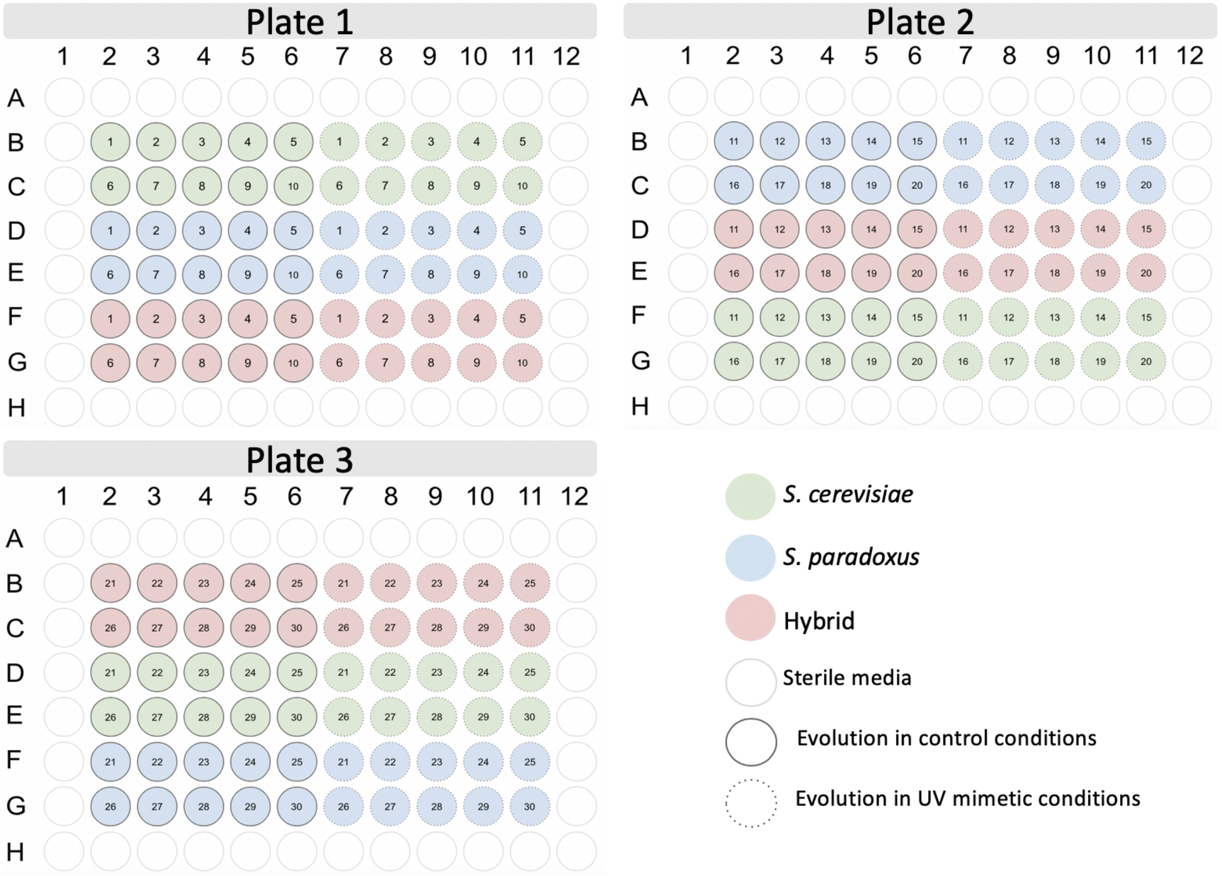

Figure S1. Design of the experiment. 30 parallel lines of each genotype were evolved under control and UV mimetic conditions in three different plates. The arrangement of the plates, with the genotypes changed position on each plate, allowed to limit the effect of position on the plate. The borders of the plates did not contain strains and were filled with sterile media to avoid border effects caused by evaporation.

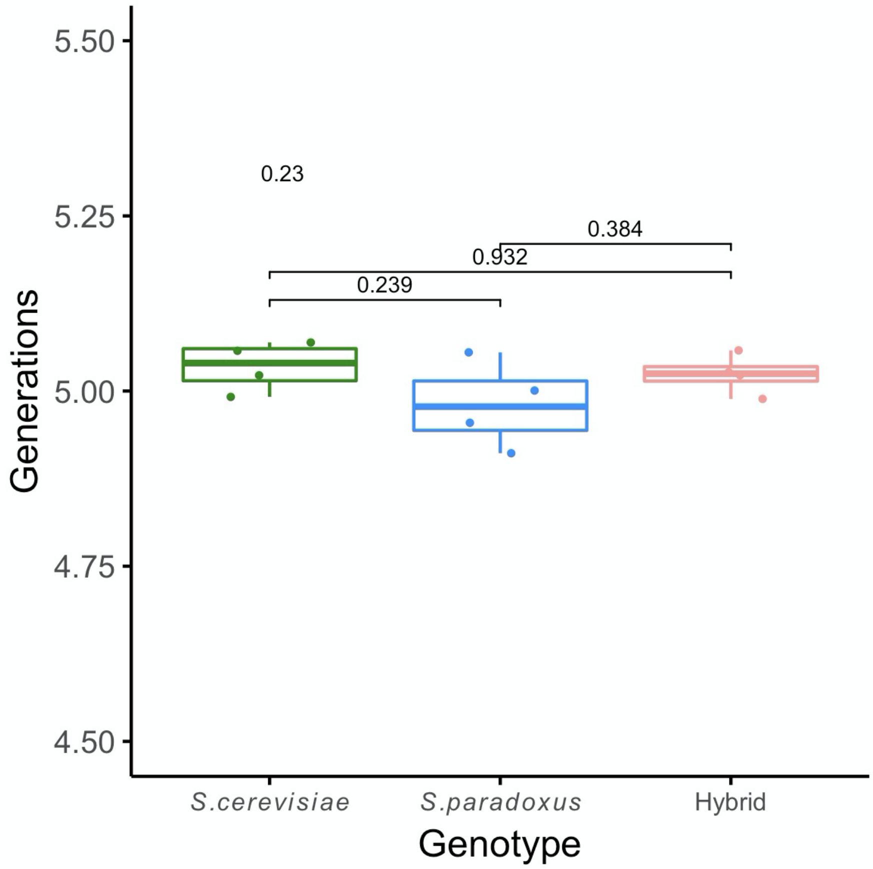

Figure S2. Number of generations. The number of generations was calculated with OD_{595} values at the beginning and the end of a cycle for the parental species and the hybrid. ANOVA and Tukey post-hoc pairwise comparisons were performed among genotypes (n = 4 populations for each genotype). P-value for ANOVA test (above) and Tukey post-hoc pairwise p-values are shown.

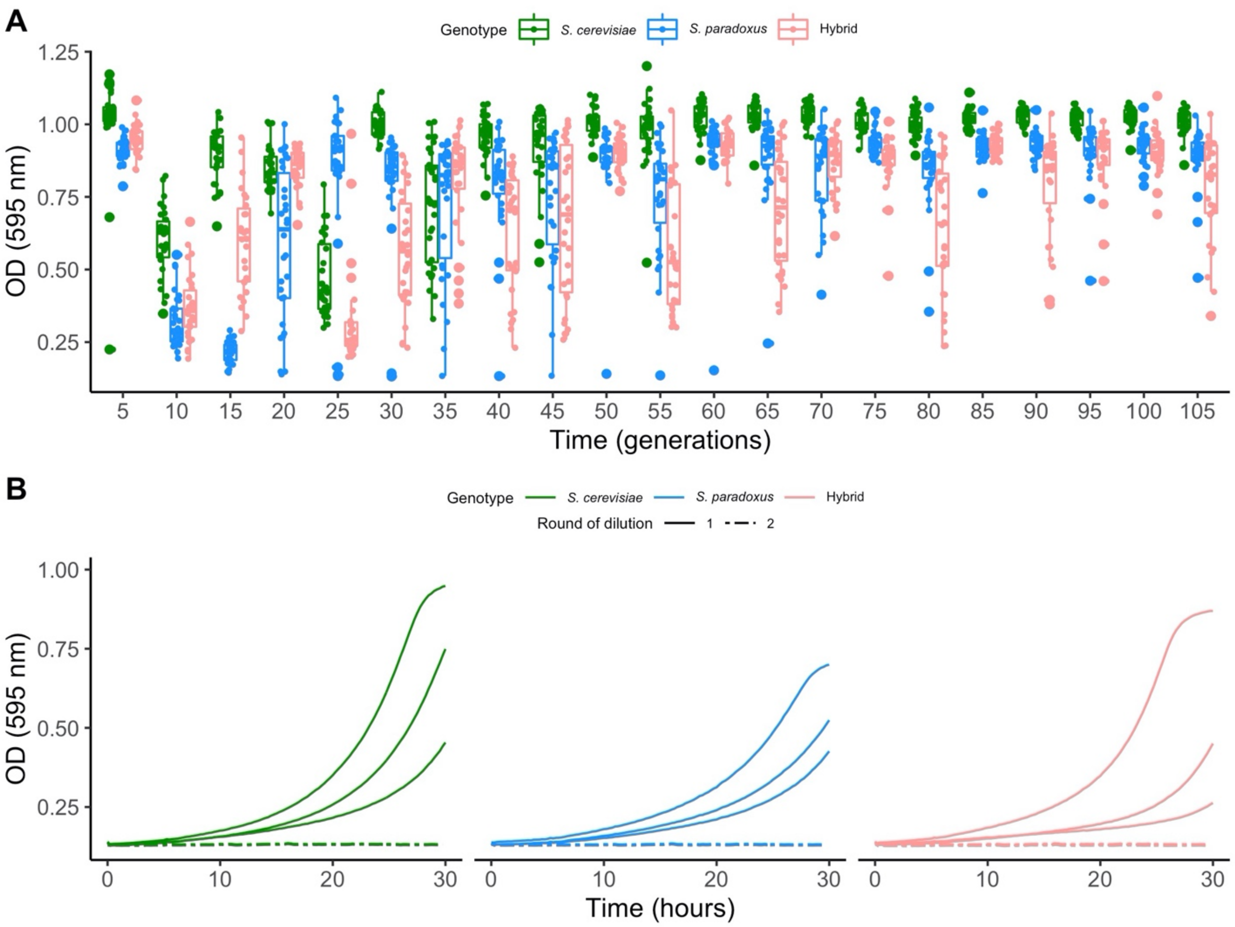

Figure S3. Cell sensitivity to the UV mimetic chemical increased during the two first cycles. A) Final optical density as a function of time (measured in generations) of each genotype in UV mimetic conditions (4 µM of 4-NQO) (n = 30 populations for each genotype). B) Optical density as a function of time (over 30 hours) of each genotype in UV mimetic conditions (16 µM of 4-NQO) (n = 3 populations for each genotype).

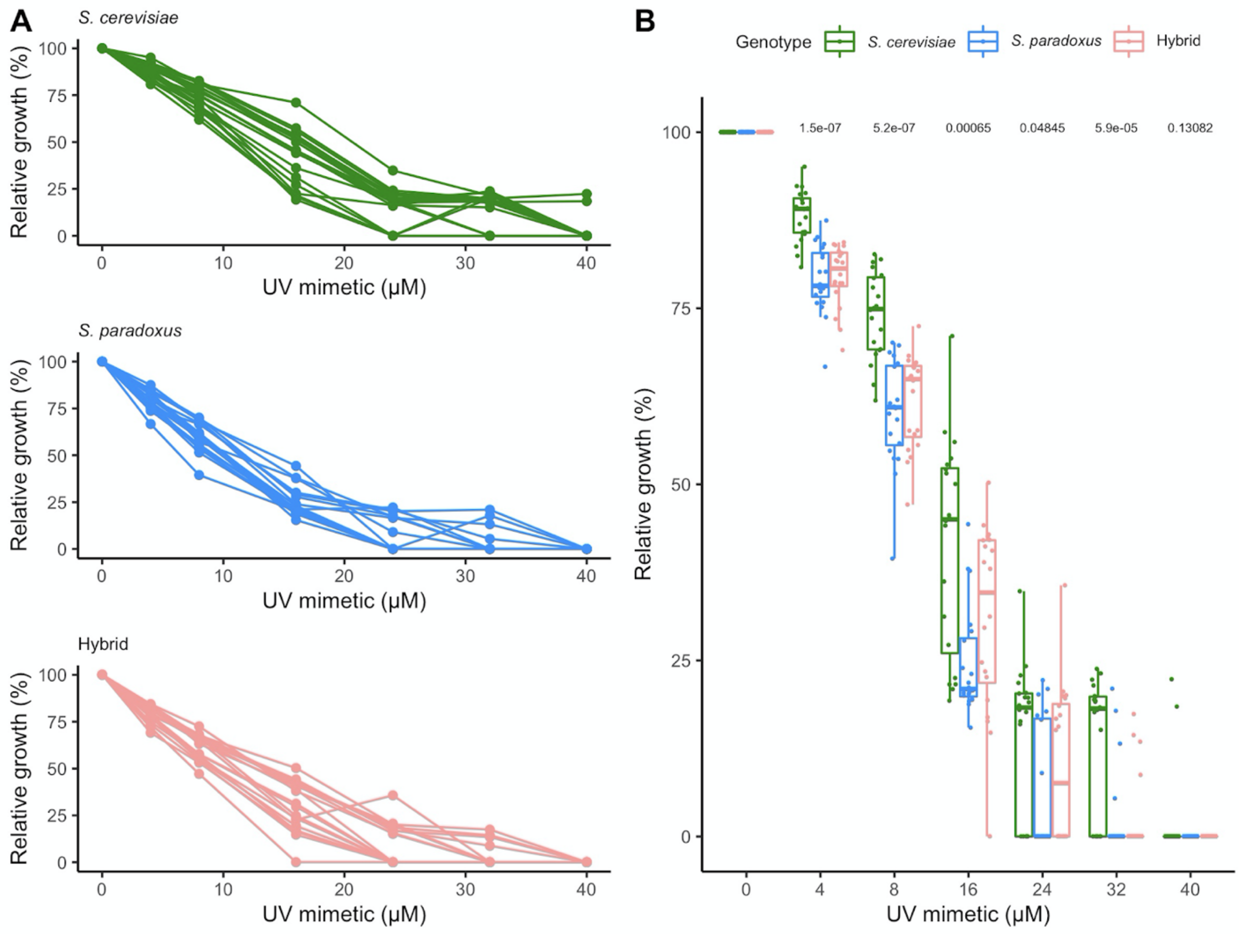

Figure S4. Effect of UV mimetic on growth. Relative growth is calculated by normalizing the growth rate in each concentration with respect to the growth rate of each strain in 0 μM of UV mimetic chemical (4-NQO) (n = 20 populations for each genotype). A) Growth of each population of each genotype in increasing UV mimetic concentrations (0 to 40 μM) B) Growth of each genotype in increasing UV mimetic concentrations (0 to 40 μM). ANOVA of the relative growth (%) of the different genotypes as a function of the concentration was performed (n = 20 populations for each genotype). ANOVA test p-values for each concentration (above) are shown.

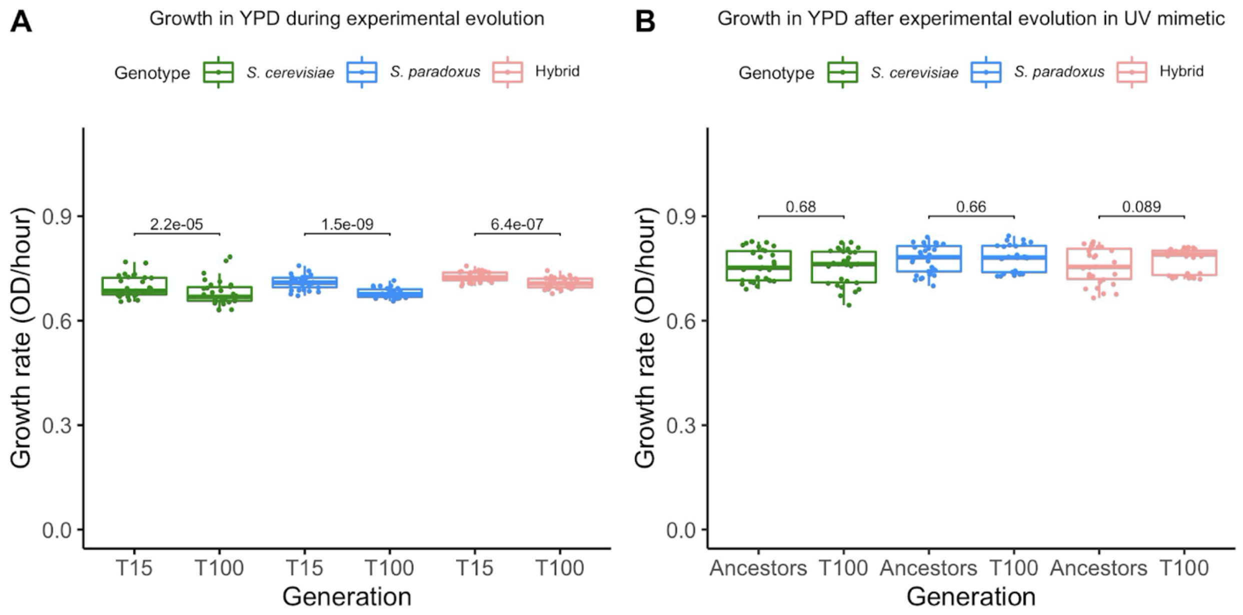

Figure S5. Growth of parents and hybrids in control conditions. A) Growth rates in control conditions (YPD) for the three genotypes evolving in UV mimetic at T15 and T100 (n = 30 populations for each genotype). Paired t-tests were performed between growth rate at 15 generations and at 100 generations for each genotype, pairing individual strains. P-values are shown (n = 30 populations for each genotype). B) Growth rates in control conditions for the three genotypes at ancestor state (from glycerol stock) and T100 (evolved in control from glycerol stock). Paired t-tests were performed between growth rate at ancestor state and at 100 generations for each genotype, pairing individual strains. P-values are shown (n = 30 populations for each genotype).

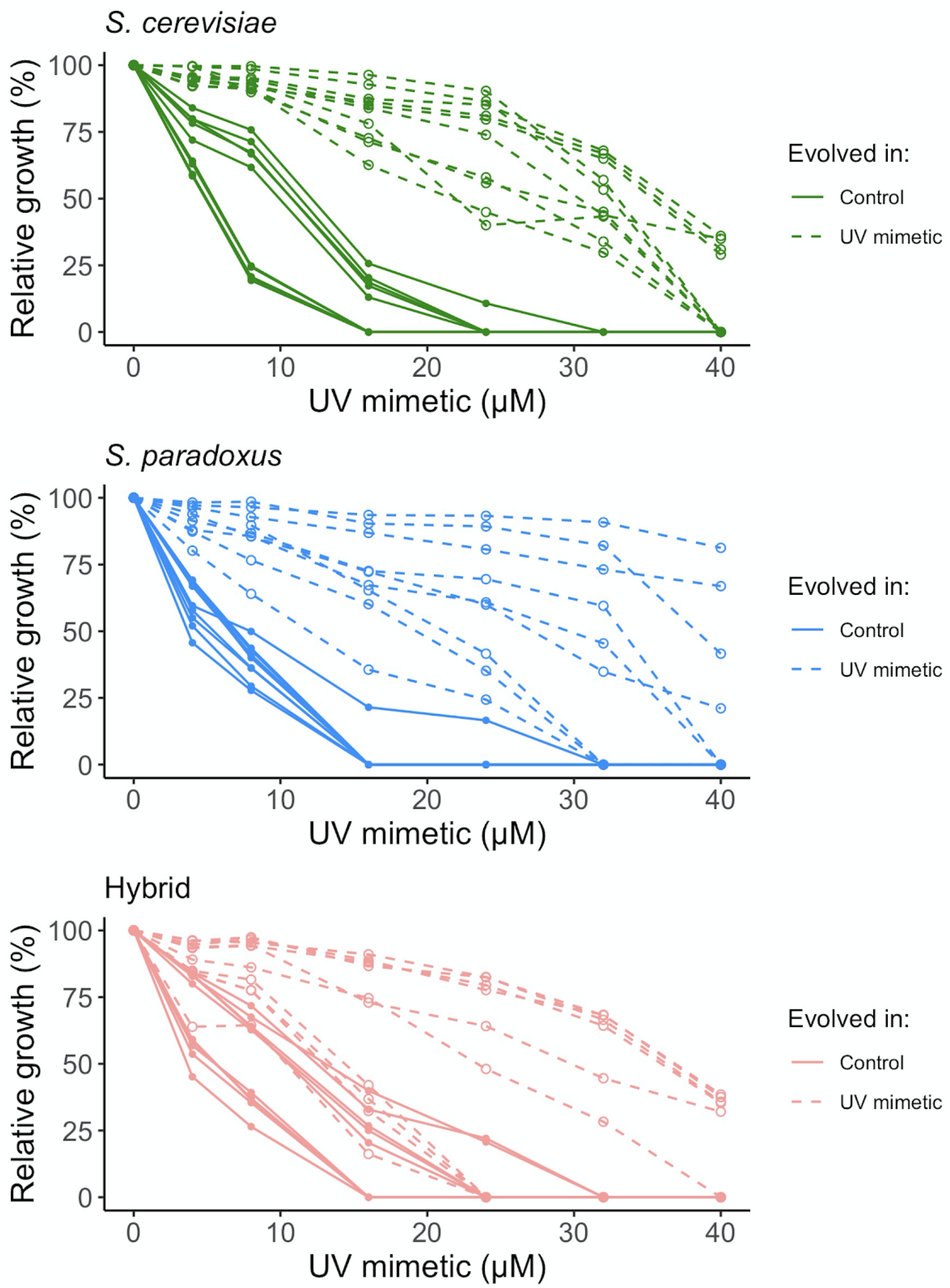

Figure S6. Adaptation to low concentration of UV mimetic leads to adaptation to higher concentrations. Growth of each population of each genotype evolved in UV mimetic or control conditions in increasing UV mimetic concentrations (0 to 40 µM) (n = 10 populations for each genotype).

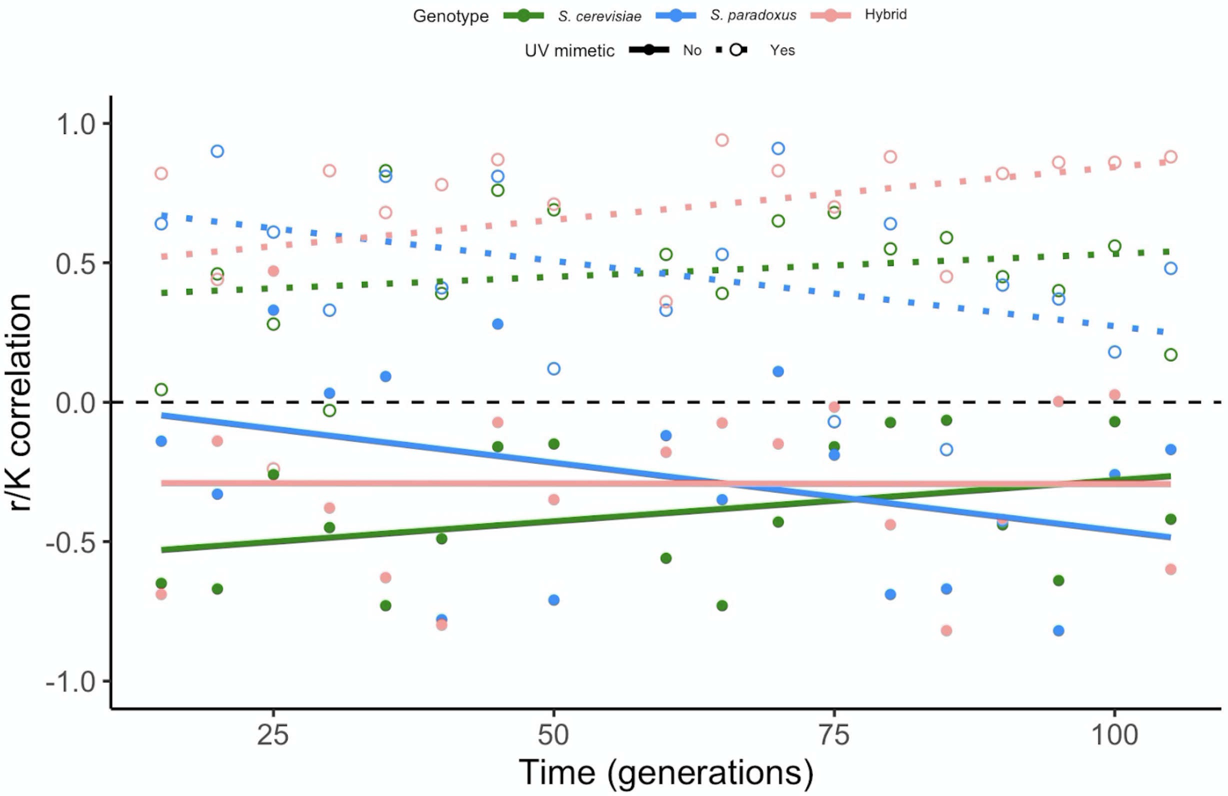

Figure S7. r/K correlations of parents and hybrids in control and UV mimetic conditions. Spearman's rank correlations between growth rate (*r*) and carrying capacity (K) through generations for populations grown in control and in UV mimetic conditions. Each point represents the value of the Spearman's rank correlation between r and K among the n = 30 populations for each genotype for a given day and a given condition.

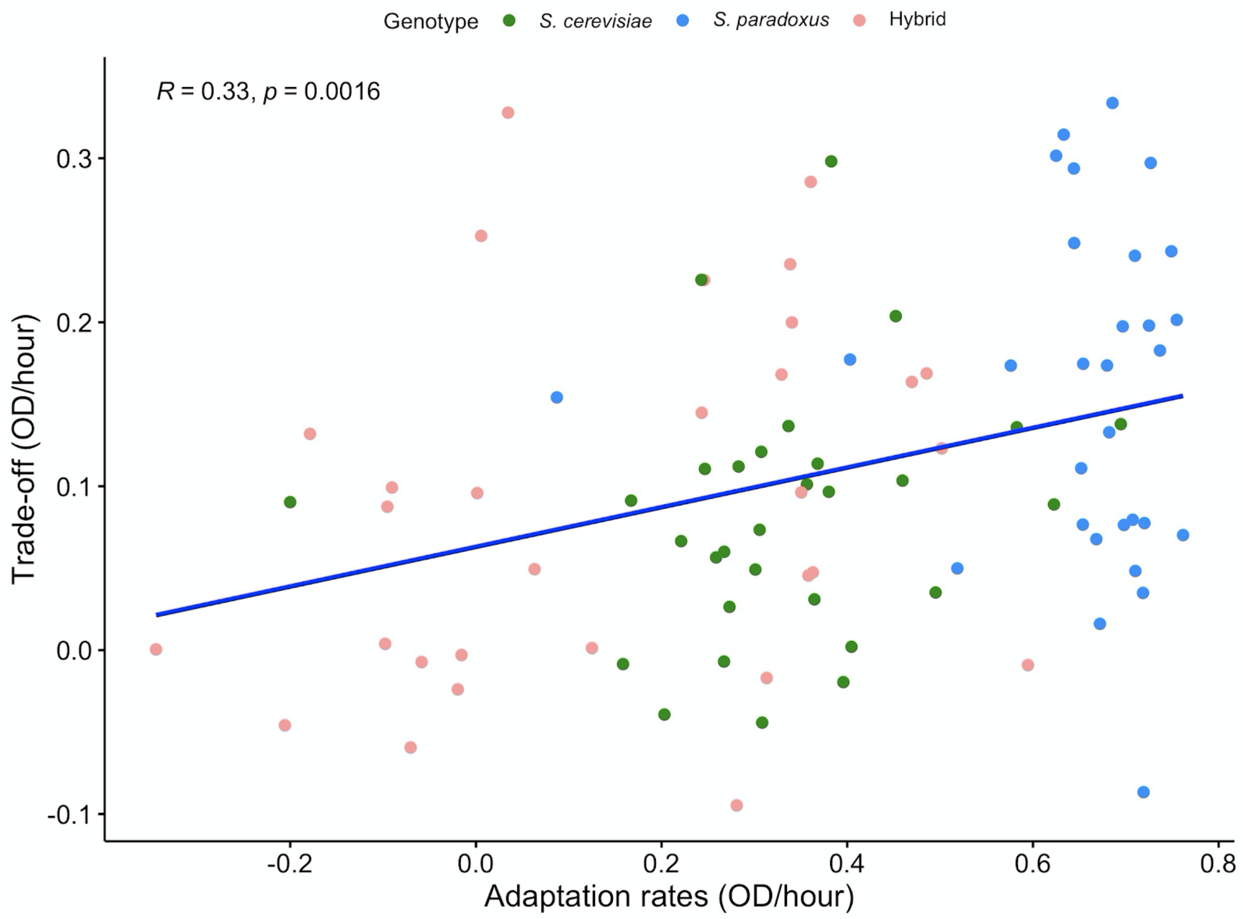

Figure S8: Trade-off depends on the extent of adaptation to UV mimetic conditions. Adaptation rates, expressed as fitness increase across the experiment, were obtained by subtracting the growth in UV mimetic conditions at T15 from the growth in UV mimetic conditions at T100. Trade-off estimates were obtained by subtracting the growth rate of the strains evolved in UV mimetic conditions (T100) grown in control conditions from the growth of the ancestors grown in control conditions. Spearman's rank coefficient and associated p-value are shown.

Table S1. List of strains used in this study.

Strain	Sampling site	Species	Mating type	Genotype	Reference Wild	Reference hoΔ
MSH604	Mont-St-Hilaire, QC	*S. paradoxus*	α	hoΔ::NAT	Leducq et al. 2014	Leducq et al. 2016
MSH604	Mont-St-Hilaire, QC	*S. paradoxus*	a	hoΔ::HYG	Leducq et al. 2014	Leducq et al. 2016
LL13_054	Rockport, MA	*S. cerevisiae*	α	hoΔ::NAT	Leducq et al. 2016	Charron et al., 2019
LL13_054	Rockport, MA	*S. cerevisiae*	a	hoΔ::HYG	Leducq et al. 2016	This study

Table S2. Models fitted with their AIC score. Three models were used to fit the adaptation in UV mimetic conditions by modeling growth rate (*r*) as a function of time and genotype.

Model	AICc	Delta_AICc	ModelLik	AICcWt
Asymptotic	-2745.955	0	1.00E+00	9.98E-01
Logarithmic	-2733.785	12.17054	2.28E-03	2.27E-03
Linear	-2656.771	89.1847	4.30E-20	4.29E-20

Table S3. Results of the linear model. "Scer" corresponds to *S. cerevisiae* and "Spar" corresponds to *S. paradoxus*.

	lm(formula = growth rate ~ generation * genotype)		
	Growth rate (OD/hour)		
	Estimate	Standard error	Pr(>|t|)
**Intercept Generation + Genotype**	0.2418973	0.0108132	< 2e-16 ***
**Generation**	0.007298	0.0008129	< 2e-16 ***
**GenotypeScer**	0.0366239	0.0152922	0.0167 *
**GenotypeSpar**	0.0044812	0.0153178	0.7699
**Generation:GenotypeScer**	0.008947	0.0011496	1.26e-14 ***
**Generation:GenotypeSpar**	0.0061001	0.0011507	1.31e-07 ***
**Degrees of freedom**	1612		
**Adjusted R-squared**	0.4353		

Table S4. Results of the Logarithmic model. The parameter "b" represents the increase in the growth rate while generations elapse for each genotype. "Scer" corresponds to *S. cerevisiae* and "Spar" corresponds to *S. paradoxus*.

	drm(rval ~ day,strain, fct = DRC.logCurve())		
	Growth rate (OD/hour)		
	Estimate	Standard error	Pr(>|t|)
**b:Scer**	0.1658994	0.0078283	< 2.2e-16 ***
**b:Spar**	0.1410741	0.0078422	< 2.2e-16 ***
**b:Hybrid**	0.075496	0.0078283	< 2.2e-16 ***

**Comparison of parameter 'b'**	
**Genotypes**	**p-value**
**Scer-Spar**	0.0252 *
**Scer-Hybrid**	6.516e-16 ***
**Spar-Hybrid**	3.968e-09 ***

Table S5. Results of the Asymptotic model. The parameter "d" is the maximum attainable for each genotype. "Scer" corresponds to *S. cerevisiae* and "Spar" corresponds to *S. paradoxus*.

	drm(rval ~ day, strain, fct = AR.3())		
	Growth rate (OD/hour)		
	**Estimate**	**Standard error**	**Pr(>|t|)**
**d:Scer**	0.625184	0.022568	< 2.2e-16 ***
**d:Spar**	0.489393	0.013855	< 2.2e-16 ***
**d:Hybrid**	0.391663	0.019385	< 2.2e-16 ***

**Comparison of parameter 'd'**	
**Genotypes**	**p-value**
**Scer-Spar**	3.286e-07 ***
**Scer-Hybrid**	7.558e-15 ***
**Spar-Hybrid**	4.307e-05 ***
